# Acute Effect of Cognitive Compromise during Physical Exercise on Self-Regulation in Early Childhood Education

**DOI:** 10.3390/ijerph17249325

**Published:** 2020-12-13

**Authors:** Nuria Ureña, Noelia Fernández, David Cárdenas, Iker Madinabeitia, Francisco Alarcón

**Affiliations:** 1 Department of Faculty of Education, University of Murcia, Street Campus Universitario, Espinardo, 12, 30100 Murcia, Spain; nuriaur@um.es (N.U.); noelia.fernandez6@um.es (N.F.); 2 Department of Physical Education and Sport, Faculty of Sport Sciences, University of Granada, 18071 Granada, Spain; ikermadi@ugr.es; 3 Sport and Health University Research Institute (iMUDS), 18071 Granada, Spain; 4 Department of General and Specific Didactics, Faculty of Education, University of Alicante, 03690 Alicante, Spain; f.alarcon@ua.es

**Keywords:** bicycle, executive functions, physical activity, cognitive implication

## Abstract

Self-regulation (SR) in pre-schoolers is a strong predictor of different aspects of mental health and wellbeing. However, SR only recently has been examined concerning physical activity and its effects on cognitive performance. In the present study, 49 preschool children aged 4–5 years were submitted to classroom movement breaks (CMBs) of 15-min with different degrees of difficulty. Before beginning the intervention, SR (i.e., head, toes, knees and shoulders test, HTKS) and skill levels were assessed for tasks demand adjustment to individual resources and the counterbalanced assignment of the participants to the groups. Similarly, after the intervention, the performance on the HTKS was re-evaluated. There was a general intervention effect on the SR of pre-schoolers, regardless of the difficulty level of the task [F (3) = 11.683, *p*-value < 0.001, η^2^*p* = 0.438]. Nevertheless, it seems that only when CMBs stimulate the children cognitively with optimal difficulty, is it possible to obtain benefits. We recommend providing teachers with professional support when implementing physical activity breaks in their daily program to generate an individualized level of cognitive load that would allow children to reach the optimal challenge point.

## 1. Introduction

The acute effects of physical activity (PA) on cognitive performance at school age have been widely studied, finding improvements in young adults as well as in children and adolescents, especially those referred to cognitive control [1]. These results would justify the convenience of increasing the levels of practice in the school environment. One of the most frequently used strategies to achieve this is the inclusion of classroom movement breaks (CMBs). However, empirical research carried out with preschool children is still scarce, so studies are needed to verify the acute effect of physical exercise (PE) on their cognitive performance and help to better understand the possible mechanisms that would justify them. Recently, interest has grown in studying the degree of cognitive compromise present in PE as a mediating factor of the possible effect. Studies that have compared the effects of an intervention based on the practice of acute PA that includes some form of cognitive compromise (attractive cognitive exercise, team games) with a non-active control condition found better cognitive performance in the experimental group [2,3]. The cognitive efforts required to develop complex or new motor skills could explain these improvements, by affecting the core processes that implement cognitive control, categorized in the domains of inhibition (i.e., the ability to ignore distractors and maintain focus), working memory (WM; the ability to hold information in mind and manipulate it), and cognitive flexibility (CF; the ability to change perspective, attention, or response mappings), which are globally called executive functions (EFs) [4,5].

At this point, it is essential to highlight that, on the one hand, the appearance of cognitive control during childhood supports greater autonomy and increasingly adaptive behaviour [6], or similarly, better behavioural self-regulation [7]. On the other hand, the correlation between EFs and self-regulation capacity has been demonstrated [8,9]. Greater self-regulation in pre-schoolers facilitates cognitive reasoning in school [10] and better performance in everyday school tasks [11].

There is a progressive differentiation of the main control components during the different stages of an individual’s life (WM update, response inhibition, task change) [12]. The correlations between measures of inhibitory capacity and WM are higher in preschool than in childhood [13]. The assumed measures of WM and inhibition seem to take advantage of the same underlying construction in preschool [14], suggesting that EFs could be a relatively unitary construction in children [15]. There is evidence that a single undifferentiated executive control factor better describes the structure of the latent EFs during early childhood and in preschool children [14,16]. This has led to the use of behavioural self-regulation assessment measures in childhood, such as the behavioural manifestation of EFs [15,16,17], through tasks such as head, toes, knees and shoulders test (HTKS).

However, developmental improvements in cognitive control can be interpreted not only quantitatively but also qualitatively [18]. The maturational development leads to an increased efficiency of these components, which would allow a more optimal control adjustment according to the objectives and demands of the related tasks [19]. This more significant adjustment can be described as a switch between two cognitive control strategies: reactive control and proactive control [20,21,22,23,24].

From this point of view, younger children would rely primarily on reactive control, which involves waiting for an event that requires control to occur and then implementing cognitive control as a late correction mechanism [25]. On the contrary, older children would tend to use proactive control, an anticipatory mechanism that involves actively keeping the information relevant to the achievement of the goal in WM, and optimally guiding behaviour when the event occurs. The developmental shift from one mechanism to another would likely occur between 4 and 6 years of age [18,22]. As children grow old, they can move from one type of control to another in a more flexible way [26]. This shift toward proactive control occurs in conjunction with age-related improvements in WM [27].

Moreover, greater childhood physical fitness is associated with superior cognitive control [28,29,30,31,32]. Previous studies found that motor skills experts performed better on executive control assessment tests compared to non-experts [33,34,35]. A relationship between motor skills and cognitive control in preschool children has recently been documented [36,37,38,39]. These cross-sectional studies are supported by longitudinal studies of a single period of PA. In contrast, in several recent meta-analyses, after analysing only high-quality studies [40] or randomized controlled experimental studies [41], sufficient evidence that acute PA has favourable effects on cognitive function in childhood has not been found. In a systematic review that analysed the acute effect of PE within the classroom (acute school-based systematic reviews of CMB studies), of the six studies that assessed cognition, only three analysed the effect on cognitive control, but none did so in a preschool population. The results did not show an improvement in cognition [42]. More recently, in the meta-analysis by Norris et al. [43] on the influence of physically active lessons in schools, no study of the acute effect of PE on cognitive performance was found at the preschool education stage.

Donnelly et al. [44] have suggested that the effect of PA on cognitive control could depend on the type of PA. Two of the conditions that could influence are the cognitive demands inherent in the structure of attractive and goal-directed exercise, and the cognitive commitment required to execute complex motor movements [45]. With this perspective, a meta-analysis differentiating the regular intervention studies (chronic effect) according to their qualitative and not merely quantitative characteristics found improvements in EFs after a PA that implied skills training, or cognitive compromise, compared to simple aerobic activities [46]. More complex movement patterns that require deeper information processing, compared to simpler patterns, produce more consistent neuroplastic changes [46].

Within the theoretical framework of embodied cognition in which the relationship between the human motor system and cognition is studied, it has been established that perception and action are closely intertwined [47]. According to Wilson [48], people learn from the interaction between their body and the physical environment and cognitive processes are based on action and perception. Neuropsychological evidence has confirmed this relationship. Structures crucial for motor and cognitive skills are activated concomitantly during motor and cognitive tasks. The cerebellum and prefrontal cortex are primarily activated in cognitive or motor tasks that are complex, unknown, require rapid reactions or underlie changing conditions [49,50]. While performing a difficult motor task, higher-order cognitive resources, such as EFs, are needed to perform the task according to its objectives [51]. The developmental literature offers several examples of the cognitive benefit of different forms of motor activities, such as the motor story, in early childhood [52]. Focused on Vygotsky’s theory [53], there are programs, such as Tools of the Mind, which suggest that inventing scenarios and acting out roles, based on dramatic or simulation play, provides children with opportunities to self-regulate and acquire responsibility [53]. Several studies have found positive results in EFs using this program [54,55,56,57,58].

On the other hand, it has been shown that, during childhood, unlike in other more advanced stages of life, walking requires attention [59,60,61], especially in preschool children [60,62,63]. This fact is even more evident when examining more complex situations, such as avoiding obstacles. In these challenging environments, children must maintain balance, so they need to continually modify their movement patterns to adjust their response to environmental limitations [64], which is known as adaptive locomotion [65]. To maintain balance in these challenging environments, cognitive control takes both a reactive strategy to deal with the unexpected disturbance and a previously planned strategy to avoid a possible disturbance in advance.

Finally, there has been little interest in analysing the interaction between physical and motor efforts to improve cognitive control during the preschool stage. In a recent review [66,67], with the aim of analysing the influence of PA on cognitive development during early childhood (0–5 years), only two studies evaluated cognitive control, although with disparate results. While Palmer and colleagues [68] found benefits in this population after 30 min of exercise, Mireau [69] found that although acute exercise induced cortical inhibition, it did not influence cognitive performance. Later, in the study by Stein et al., [70] children who were randomly assigned to the intervention condition did not show an improvement in performance on EFs tasks relative to children in the control condition. To control the order effect, the evaluation tasks were randomized. This allowed finding a positive effect when the first task was the assessment of inhibition, although only in it and not in the rest of the tasks. In all of them, the PE tasks had coordinative motor demands. The possible causes could fall on the lack of control of the task complexity level, both of the experimental task (the one that generates the possible changes in the children), and in the evaluation task (in which the effects are perceived). The cognitive control necessary to respond to the task in which cognitive performance is assessed will depend both on the nature of the task and the level of the children’s motor and cognitive functions [71]. Therefore, two types of difficulty can be distinguished [72].

On the one hand, the nominal difficulty, which is related to the characteristics of the task without taking into account the characteristics of the individual and would depend on the number and complexity of its elements. On the other hand, on the human side, the difficulty or workload experienced depends on the resources available to the person to face the requirements of the task [73]. In this sense, the functional difficulty or undesirable entropy reflects the inadequacy of the person to face the uncertainty of the environment and represents the level of the task considering the capacities of the individual and the conditions of the context [74]. Thus, for a skill level and the same context, functional difficulty depends on the nominal difficulty of the task, but also on the capacities of individuals to process information. Both types of difficulty determine the cognitive-motor responses [72]. Unfortunately, a major review of the literature shows that this type of control did not take place in most of the initial investigations. Moreover, although entirely different activities are compared in most studies [74], any comparison of the effect of different degrees of complexity on subsequent performance has been made. Additionally, the difficulty of the tasks perceived by each individual should be considered. According to Maurer et al. [73], one of the possible causes of the lack of positive results could be the accumulation of mental fatigue produced by the high cognitive demands of the experimental and the evaluation tasks. This accumulated mental load could reduce the cognitive resources to solve the following evaluation tasks, especially considering the few resources available in the preschool population. However, this possible damage also depends on the cognitive demands of these tasks. To the best of our knowledge, no previous study has taken this fact into account. Different results could be obtained according to degrees of nominal difficulty of the tasks in which the effects are going to be measured.

Thus, the present study aimed to verify the effect of manipulating the nominal difficulty of the tasks on the capacity for behavioural self-regulation of pre-schoolers, trying to keep the functional difficulty of the task as constant as possible, by controlling the level of motor skill from the start of the participants. The authors hypothesized that, in these conditions, physical activity tasks with the presence of cognitive requirements would improve cognitive control and self-regulation.

## 2. Materials and Methods

### 2.1. Design

A group-randomized controlled trial was performed to test the acute effect on behavioural self-regulation using CMBs with different degrees of difficulty. Before beginning the intervention, behavioural self-regulation (i.e., HTKS), and cycling skill levels (i.e., general dynamic coordination) were assessed for tasks adjustment to individual demands and the counterbalanced assignment of the participants to the groups. Since in the school setting, the random assignment of individuals to different experimental conditions is not feasible, randomization of the groups was used for the ecological validity of the school classroom settings. First, the classes were assigned to the groups with and without locomotion. Randomization by blocks of the participants of the locomotion class was carried out depending on the cycling performance. For the class without locomotion, simple randomization was performed. Thus, the 49 participants were assigned to 4 groups: a group of Locomotion with Bicycle (BL; twelve participants, seven girls); a group of Locomotion Without Bicycle (WBL; thirteen participants; 9 girls); Dramatic Story group (DS; twelve participants; 9 girls) and control group (CG; twelve participants; 8 girls). Similarly, after the intervention, the performance on the HTKS was re-evaluated.

### 2.2. Participants

Forty-nine preschool children aged 4–5 years [(M_age_ = 54.38 (±3.49); 1 with Attention-Deficit/Hyperactivity Disorder (ADHD) and 1 immigrant] formed the sample. Due to the characteristics of the study, the selection of the sample was made in a non-probabilistic way and for convenience. The participants belonged to two classrooms on the third level of the Second Cycle of Early Childhood Education. All participants’ families signed the informed consent form approved by the Ethics Committee on Human Research of the University of Murcia, Spain (ID: 2916/2020) and were treated under the Helsinki declaration (2013) [75].

### 2.3. Procedure

**Figure 1:**
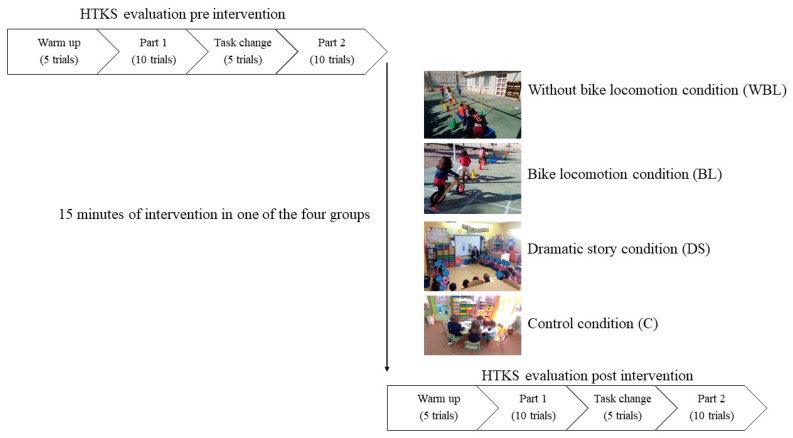
Procedure of the study.

Each of the four groups (3 experimental and one control) underwent an intervention that involved different tasks, lasting 15 min. Children were instructed to participate in games with increasing difficulty, specifically designed to stimulate EFs. The motor tasks were carried out during school hours, such as CMBs, and consisted of the same content for each group, varying the amount of cognitive involvement and the use or not of the bicycle without pedals. In Figure 1 is depicted the procedure of the study. Additionally, Table 1 describes each of the experimental conditions, taking into account the proposal of Schmidt et al. [76]. The three experimental conditions are presented through two characters named Migo and Miga and contextualized through symbolic games.

#### 2.3.1. Without Bike Locomotion Condition (WBL)

A circuit with obstacles was designed (cones to be overcome in zig-zag, a tunnel with spikes, a ramp with mats and ropes to pass under without touching them), and an obligation to maintain balance through delimited spaces (go over letters painted on the ground). The tour was carried out three times.

#### 2.3.2. Bike Locomotion Condition (BL)

This group performed the same circuit as the WBL group but riding on a non-pedals bicycle. The use of this implement increases the demands of dynamic balance, increasing the nominal difficulty of the task.

#### 2.3.3. Dramatic Story Condition (DS)

A dramatic story was used [55] in which children’s literature and body movement are combined. Participants had to use body movement to act out an imaginary story guided by the teacher. The tale demanded the continuous accompaniment of the instructor for the development of the plot of the story and the movements. The task was carried out in the classroom, with the participants seated while the teachers read the story “Mystery in the castle of the cycling witch”.

#### 2.3.4. Control Condition (C)

The task was carried out in the classroom, with the participants seated at worktables. They were given a sheet with the characters from the dramatic tale called MIGO and MIGA to be painted, leaving a model of the characters on the wall, in an area with good visibility.

### 2.4. Measures

#### 2.4.1. Self-Regulation

The Spanish version of the head, toes, knees and shoulders test (HTKS) was used to assess self-regulation [77].

The extended version of this task is a measure of behavioural self-regulation that requires CF, WM, and inhibitory control [7,78]. The HTKS has been used with a large number of children and has good construct validity and reliability [78,79,80]. The task requires different components of EFs such as inhibitory control, by making the child pay attention to the rules in which a natural response must be inhibited, and the opposite of command must be executed. Additionally, assessment requires WM and attentional changes, and previous research has found that the task is related to all aspects of EFs [81,82]. To complete the HTKS, children must do the opposite of what the examiner says, for example, when children are asked to “touch their toes,” they touch their heads, and vice versa. Therefore, children must not only retain task rules in memory but also inhibit the tendency to give a natural, otherwise correct response (e.g., avoid touching their toes when they are told “touch your toes”), while responding oppositely (e.g., touch your head). It also involves increasing the cognitive demands because the rules of the task become more complex, by adding a new set of commands (“touch your shoulders”, “touch your knees”).

The HTKS is divided into two parts, taking approximately 3–5 min to be completed. Part I consists of 5 familiarization and 10 test trials, during which children respond to one of the two paired commands (“touch your head” or “touch your toes”) with the opposite response. Each wrong answer is coded 0, a self-correcting answer is coded 1 point, and a correct answer is coded 2 points. Self-correction is defined as any movement aimed at the wrong answer but ending with the correct action. In part II, two additional commands were added (“touch your shoulders” and “touch your knees”). After completing 5 practice trials with only the new commands, children complete 10 test trials in which they have to respond to all four commands. Taking into account that the addition of this new rule could demand the capacities of CF of the sample, we included the performance of the 5 first practice trials of the second part of the test in the analysis as a variable that could assess the possible effect of task change (TC). In summary, there is a total of 30 items in the whole test with a possible scoring range from 0 to 60 (for a further explanation of the test procedure, consult [83,84]). Before carrying out the evaluation using the HTKS, the researcher underwent training through a pilot study, in order to improve her skills in using the instrument.

#### 2.4.2. General Dynamic Coordination

From the battery validated by Carmona [85], the test for general dynamic coordination has been extracted and has been adapted to include and evaluate coordination and mastery of the bicycle without pedals. The purpose was to measure reaction speed, travel speed, and agility in general terms with the bike without pedals measured in seconds and tenths of a second. Specifically, the test consists of showing the child a table tennis racket which colour corresponds to the cone she has to go. The time of the three attempts is added.

#### 2.4.3. Cognitive Control Estimation

As mentioned above in the behavioural self-regulation test, one part of the performance score of the HTKS is attributed to self-corrected responses with one point. One of the hypotheses of this study is that PE might provoke that individuals improve their self-regulation behaviour changing their reactive cognitive control to a proactive control. Thus, it is plausible to consider that the number of self-corrected responses could be significantly different between pre- and post-evaluations. Nevertheless, the scoring system of the HTKS test may not be sensitive enough to analyse this effect. For example, both participants “A” and “B” have one self-corrected pre-intervention response and zero self-corrected post-intervention responses. However, the two participants differ in that participant “A” has one more correct post-intervention answer and participant “B” has one more incorrect post-intervention answer, which could mean that participant “A” was capable of transforming the self-corrected answer to a better one. In contrast, participant “B” failed and achieved a worse result. In this regard, it is clear that participant “A” had a better self-correction quality rather than “B”, but the scoring system is not sensitive to these changes, so a new scoring system has been elaborated to approach the authors’ hypothesis, named cognitive control estimation (CCE): (1) 1 point for each self-corrected pre-intervention answer that will transform into a correct post-intervention answer; (2) 1 point for each incorrect pre-intervention answer that will transform into a correct post-intervention answer; (3) −1 point for each correct pre-intervention answer that will transform into a self-corrected post-intervention answer; (4) −1 point for each correct pre-intervention answer that will transform into an incorrect post-intervention answer.

#### 2.4.4. Statistical Analysis

Data summaries were computed for the whole sample. Firstly, a Shapiro–Wilk normality test was conducted for the whole variables of interest to use parametric and nonparametric tests where appropriate. Secondly, to verify that there were no significant differences in general dynamic coordination between WBL and BL, a Mann–Whitney test was performed. Thirdly, to observe the group effect in the HTKS test, pre- and post-intervention scores were submitted to a Wilcoxon test. Lastly, to establish significant differences between groups, a differential score (post- minus pre- score) of both HTKS tests was calculated and submitted to an ANOVA, while CCE was submitted to a Kruskal–Wallis test. Separately, gender was included in each analysis to observe whether it could influence the performance of the tests.

The level of significance was set at 0.05, and the Bonferroni correction for multiple comparisons was used where applicable. The standardized effect size was reported, employing the partial η*p*^2^ for Fs and d in post hoc analysis and *t*-tests, and r for the Wilcoxon paired-sample test, of which the formula is z/√(*n*) where z is the z-statistic and *n* the number of observation. Partial η*p*^2^ is based on Cohen’s f, which defines small, medium and large as respectively 0.10, 0.25 and 0.50, which corresponds to η^2^ of 0.0099, 0.0588 and 0.1379 [86], and both d and r use the Cohen’s interpretation guidelines of 0.1 (small effect), 0.3 (moderate effect) and above 0.5 as a strong effect [87,88]. The JASP statistics package (version 0.8.1.2, JASP team, Amsterdam) was used for the analysis conducted.

## 3. Results

Means and standard deviations for each variable of the study are displayed in Table 2. The Shapiro–Wilk test showed that only the differential scores variables were normal, while the others did not follow a normal distribution. Moreover, there were no significant differences in general dynamic coordination between WBL and BL (Z = −0.218, *p* = 0.828), and there were not any significant differences in the following tests when the variable gender was included.

### 3.1. Self-Regulation

**Figure 2:**
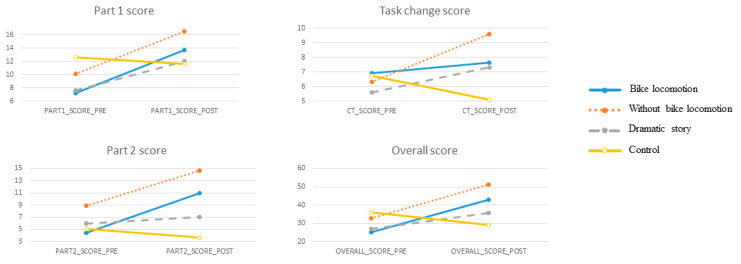
Group effect pre-post score in the different parts of the head, toes, knees and shoulders (HTKS) test.

Group effect: Concerning HTKS test, Wilcoxon paired-sample test revealed that the BL group improved significantly in the part 1 score (Z = −2.407; *p*-value = 0.016; r = −0.695), part 2 score (Z = −2.293; *p*-value = 0.022; r = −0.662) and overall score (Z = −2.845; *p*-value = 0.004; r = −0.821). The WBL group has also shown a significant improvement in part 1 score (Z = −3.066; *p*-value = 0.002; r = −0.851), TC score (Z = −2.952; *p*-value = 0.003; r = −0.818), part 2 score (Z = −2.806; *p*-value = 0.005; r = −0.778) and overall score (Z = −3.181; *p*-value = 0.001; r = −0.882). DS group results showed had a significantly better performance in part 1 score (Z = −2.106; *p*-value = 0.035; r = −0.607) and overall score (Z = −2.584; *p*-value = 0.011; r = −0.745). Lastly, no significant results were observed in the control group. These results are visually represented in Figure 2.

Between groups analysis: ANOVA tests revealed that there were significant results in the differential scores (i.e., post- minus pre- scores), TC [F (3) = 3.063, *p*-value = 0.038, η^2^*p* = 0.171; post hoc showed that WBL improved significantly better versus control group (t = 2.962, *p*-value = 0.029, d = 1.078]; part 2 score [F (3) = 4.846, *p*-value = 0.005, η^2^*p* = 0.244; post hoc again showed that both BL and WBL groups improved significantly better versus the control group (t = 3.252, *p*-value = 0.013, d = 1.204; t = 2.979, *p*-value = 0.028, d = 1.422)] and overall score [F (3) = 11.683, *p*-value < 0.001, η^2^*p* = 0.438; post hoc revealed that BL, WBL and DS groups improved significantly better versus the control group (t = 4.962, *p*-value < 0.001, d = 1.831; *t* = 5.288, *p*-value < 0.001, d = 1.923; *t* = 3.123, *p*-value = 0.019, d = 1.254)]. These results are shown in Figure 2.

### 3.2. Cognitive Control Estimation

**Figure 3:**
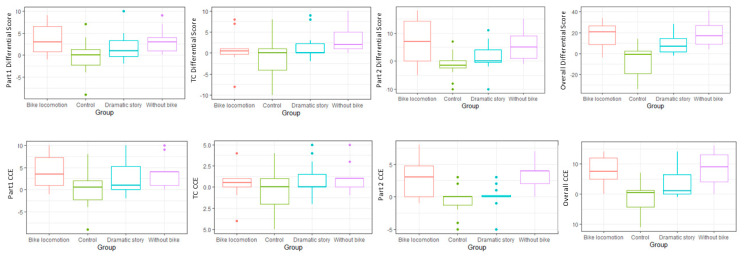
Differences in the group effect of the self-regulation (upper part of the panel) and cognitive control estimation for each part of the HTKS test.

Concerning CCE, the Kruskal–Wallis test showed significant results in part 2 [χ^2^ (3) = 20.71, *p* < 0.001; post hoc analysis revealed that both BL and WBL groups had a better CCE versus DS group (Z = −2.284; *p*-value = 0.022; r = −0.466; Z = −3.553; *p*-value < 0.001; r = −0.711) and control group (Z = −2.774; *p*-value = 0.006; r = −0.566; Z = −3.682; *p*-value < 0.001; r = −0.736]. Significant results were also observed in overall score [χ^2^ (3) = 18.587, *p* < 0.001; post hoc analysis revealed again that both BL and WBL groups had a better CCE versus DS group (Z = −2.035; *p*-value = 0.042; r = −0.415; Z = −2.435; *p*-value = 0.015; r = −0.487), and control group (Z = −3.332; *p*-value = 0.001; r = −0.681; Z = −3.571; *p*-value < 0.001; r = −0.714)]. Lastly, marginally significant differences were observed in part 1 [χ^2^ (3) = 7.474, *p* = 0.058]. These results are also represented in Figure 3.

## 4. Discussion

The current study is aimed at verifying the acute school-based PE effects on behavioural self-regulation in a sample of preschool children. Unlike previous studies, we manipulate the nominal difficulty, establishing three levels of complexity. As expected, there was a general effect of the intervention on the self-regulation of pre-schoolers, regardless of the nominal difficulty level of the task, reproducing the beneficial effect found in school populations of the PE with cognitive involvement [1,40] or through strategies such as dramatic play or role-play [56,57,89]. The three intervention groups managed to improve the performance on the HTKS, unlike the control group, which did not significantly change. Both dramatic play and coordinative tasks with aerobic demands had acute benefits in children’s self-regulation. These results are in line with studies reporting the positive effects of coordinative interventions and aerobic exercise interventions on motor inhibition in kindergarten children [75]. However, other studies failed to find effects of acute aerobic exercise or coordination interventions on EFs in this population [67,69,75]. These contradictory findings could be attributed to the differences both in the type of physical task and in the tasks used to assess EFs.

The most important result was, however, how each experimental condition influenced the different parts of the HTKS test. In the first part of the test, in which there was only one rule, considering the overall results, the three experimental groups improved their performance. On the other hand, in the second part of the test, in which the participants had to implement a new rule, in addition to maintaining the previous one, the dramatic-play group stopped obtaining benefits, something that the two groups of PE with coordinative demands did. Similarly, these two groups also improve their CCE. Apparently, it might seem that both measures could be evaluating the same thing, but the fact that in the first part of the test the performance of the three groups was similar and varied only in the second part, when the level of difficulty was higher, allows intuit that the differences may be due to the fact of having changed the type of cognitive control. In other words, the cognitive control estimation index has made it possible to understand the nature of the change in self-regulatory behaviours.

A possible explanation can be found in the “challenge point framework” proposed by Guadagnoli and Lee [90]. The authors state that subjective difficulty is crucial to the learning process. The dramatic story practice situation may not have provided an adequate amount of task difficulty to facilitate cognitive processes. It would be necessary to exceed a minimum threshold of stimulation to find benefits [69]. After reviewing the programs that influence EFs, Diamond and Ling [89] established that in order to find differences between the treatment and control groups, the tasks should require participants to use their cognitive skills close to its limit. The most remarkable differences between the groups are consistently found in the most demanding EFs tasks [58]. This threshold may be related to the change in the type of cognitive control. Children may have a higher threshold for participating in proactive monitoring than adults. Given the relatively underdeveloped nature of their proactive control, children can use it only when reactive control is much more difficult to be implemented. Chevalier [18], using the cost of changing tasks, found that while 10-year-olds take control proactively whenever possible, 5-year-olds only do so if reactive control becomes more difficult. Children tend to be overly dependent on reactive control, despite both the fact that from 5 years of age they are already able to participate proactively when encouraged to do so, and they function better when using it [21,91,92]. The HTKS test requires solving a conflict between two possible answers, having to inhibit the preponderant one. Reactive stopping requires completely countermanding the initiated response.

On the contrary, proactive control could inhibit the incorrect response before the possible responses are activated [93], being a more effective mechanism when active information is available in the WM. A self-correcting response on the part of the child during HTKS could be explained by reactive inhibition. Upon launching the answer, the child realizes and corrects their decision, albeit late. In this way, a decrease in self-correcting responses after experimental tasks could indicate a change from reactive to proactive control. As in the most difficult task, the groups that performed physical and coordinative exercise obtained benefits in this variable, reducing the number of self-corrections. Both results could indicate that children who performed physical and coordinative exercise could stimulate a change from reactive to proactive control. Increased infant fitness is associated with a more flexible switch between reactive and proactive modes of cognitive control [94].

Regarding the cost of the task change, only the locomotion condition without a bicycle improved the results in the post-test. The beneficial post-exercise effects on self-regulation of the cycling locomotion group may have been nullified due to fatigue caused by the additional cognitive demands during cycling [95]. The cognitive and attentional resources of kindergarten children are more limited than those of older children [96]. Therefore, the execution of a demanding cognitive task could reduce the cognitive resources for the next task [75]. WM decreases as cognitive load increases. Multicomponent tasks could cause a cognitive overload that hinders the control of executive attention [97,98,99].

In this study, the children who performed the task with a bicycle could have been assuming a great functional difficulty that could have generated specific cognitive fatigue. As explained [1], neural resources are used to a greater extent to regulate behaviours of a physical nature. The physical tasks and coordinative demands of this type of intervention could be competing for the same volitional resources available [100]. These demands, generated by PE, would require the participation of the same control processes involved in the coordinating tasks used in the research. For example, in young adults, changes in attentional resource allocation (as indicated by P3 event-related brain potential) after a cognitively engaging exercise episode were found to be attenuated relative to aerobic physical activity on a treadmill with equivalent cardiovascular intensity, but slightly elevated relative to rest [101]. The constant regulation of effort in a more demanding environment caused by the greater demands for cognitive control in the double physical-cognitive task could be the cause of the lack of benefits, according to the authors.

The type of cognitive mechanism may determine the acute effect of a PE. It seems logical to think that, just as resistance training produces deterioration in the ability to perform muscle contractions at the end of a training session, the demand for particular cognitive abilities above a determined and individual stimulation threshold of each person, will cause a deterioration of cognitive performance, due to the depletion of available resources. There are indications that the body’s response to stimuli of a cognitive/emotional nature could follow a similar pattern to that manifested after being subjected to stimuli of a purely physical nature [74]. It means that its deterioration would be conditioned by exceeding a certain threshold and its improvement by allowing the time necessary for the body to be able to mobilize the necessary adaptive resources.

Nevertheless, the present results must be considered, taking into account some limitations. On one hand, the components of each group were distributed according to their group class, and not randomly. On the other hand, the sample of each group was not sufficiently large to make strong claims about the effects observed. Finally, although the authors would like to highlight that it could be a good start point, the CCE was calculated through a test (i.e., HTKS test) that was not explicitly designed to assess cognitive control. Thus, it is necessary that the design of future studies of cognitive control use tests that are sensitive enough to evaluate possible variations of cognitive control and observe if their results are in concordance with this study.

Furthermore, future interventions should be tailored to generate an individualized level of cognitive load that would allow children to reach the optimal challenge point (which represents the degree of functional difficulty of the task for each child [90]). Indeed, complexity in the motor task that deviates from the child’s initial response level could harm her later cognitive performance. Therefore, the assessment of the abilities of each child must be taken into consideration when designing any intervention proposal [75]. According to Rueda and Posner [102], one of the keys is to have external control of the cognitive difficulty of the task that is adjusted at all times to the real capacities of the person. On the other hand, in most studies, the mean values have been compared between groups, ignoring individual gains. The results between participants after training sometimes differ significantly [103]. This precedent leads us to think about the importance of studying individual differences and gains during sessions to understand these differential results [104]. Thus, when children exercise at their specific skill level, a beneficial effect on cognitive function may be more likely [105].

## 5. Conclusions

In summary, this study has observed how PE with cognitive involvement [1] improves self-regulation and cognitive control in pre-schoolers. Due to the critical role of self-regulation capacity on the scholars’ behaviour, this study supports the introduction of physical movement breaks in the school. Nevertheless, it seems like only when PE stimulate the children cognitively over a minimum threshold is it possible to obtain benefits.

The results showed that according to the task change, only the group practising the WBL condition improved the results after the intervention. Similar to the fitness setting training over a threshold, which represents the number of available resources, overcome the organism capacity to giving an adaptive response, and as a consequence, performance worsens. Based on this interpretation, further research should explore not only the influence of nominal, but also the functional difficulty of the tasks. It would be interesting to test whether this acute negative effect could be a reflection of sufficiently stimulating the organism to cause chronic effects.

## Figures and Tables

**Table 1:** Description of experimental conditions.

**Experimental Conditions**	**Description**	**Executive Function Involvement**
**-Without Bike Locomotion Condition (WBL)** An obstacle circuit was elaborated. The children had to go through the enchanted forest to save our friends Migo and Miga. 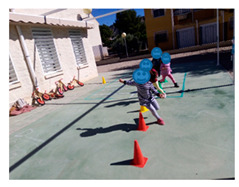 **-Bike Locomotion Condition (BL)** It is the same as the WBL group, although the children had to perform the circuit with a bike without pedals. 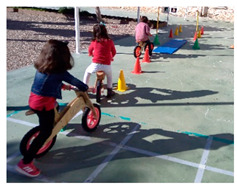	The circuit was divided into the following sections: Forest: in a delimited area, coloured cones were placed in a row, to zigzag without touching them. Tunnel: next, a tunnel with spikes for displacement, and a ramp with inclination was placed. Bridge: there was a coloured ribbon on the ground with different shapes, and they have to pass over in balance. Ponds: next to it, several disordered blocks will be placed, in order to pass between them without touching them. When they reached the final cone, they turned around and made the same circuit backwards. The circuit was performed three times. The difficulty was increased by adding more prevailing behaviour inhibition demands. In the second round, the teacher could change the information regarding each station. For example, when they got to the forest, the teacher said to go to the tunnel section. In the third round, a new rule called witch rule was included. If the teacher said this word, all the children should stop and go back to the beginning of the circuit.	Remember the rule associated with the material; adapt to rule changes, remember different rules and motor actions; facing with interfering distractors (such as other children’s movements); inhibit predominant motor responses; give a flexible motor response to the motor challenges of the circuit through adaptive locomotion: Plane changes in the horizontal and vertical axis, dynamic and static balance; Self-control of impulsivity towards the demands of the order of the sequences and order of the children in the rows; Emotional self-regulation after motor or rules errors. In the BL condition, the increase in nominal difficulty has a direct impact on adaptive locomotion requirements; more outstanding dynamic balance; greater coordination needs. Furthermore, an indirect repercussion: these attentional demands compete with the demands of the circuit which increases the demands of cognitive control, being able to decrease the executive attention to the circuit, and increasing the needs of emotional self-regulation by mistake.
**Dramatic Story Condition (DS)**	**Description**	**Executive Function Involvement**
-Reading and dramatization of the story “Mystery in the cycling witch’s castle”. 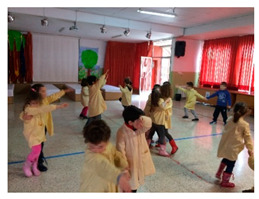	Execute the dramatization by doing as if they were the characters according to the story of the narrated story (example): *However, Migo and Miga had to be very careful, because it was not easy to get to that place, and we have to move very slowly so that the little monsters cannot see us (we move slowly through space).* *To get to the fantastic world, you first have to cross the field of the little monster Bicifoot (we pretend we go through the field on tiptoe so as not to make noise).*	Deal with interfering distractors (movements of other children); Give a flexible motor response to the motor challenges of tasks through adaptive locomotion: dynamic and static balances; self-control of impulsivity towards task demands (standing still and performing specific motor action).
**Control condition (C)**	**Description**	**Executive function involvement**
Paint MIGO and MIGA characters 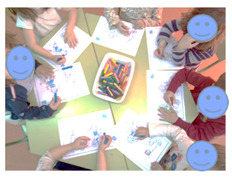	Paint sheet of the two characters Migo and Miga. 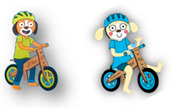	To inhibit predominant responses (Paint according to the colours established in the model sheet).

**Table 2:** Descriptive statistics for the variables of the study.

Variable	Bike		Bikeless		Acting			Painting
	Mean	SD	Mean	SD	Mean	SD	Mean	SD
Age (months)	53.92	2.99	54.54	3.92	55.51	3.47	53.58	3.63
GDC	39.01	17.83	39.33	24.18	-	-	-	-
Warm-up PRE	7.08	4.51	8.54	4.41	8.25	5.21	11.58	0.51
Part 1 PRE	7.17	8.08	10.08	7.59	7.58	7.61	12.67	6.91
TC PRE	6.92	3.84	6.31	3.98	5.58	4.52	6.75	4.11
Part 2 PRE	4.42	5.11	8.92	6.61	5.92	6.24	5.08	5.48
Overall PRE	25.17	14.186	32.62	16.09	27.17	20.68	36.00	12.00
Warm up POST	10.42	2.712	10.54	2.22	9.33	4.92	8.75	5.08
Part 1 POST	13.75	7.83	16.54	5.33	12.00	8.12	11.67	8.37
TC POST	7.67	3.65	9.62	1.61	7.33	4.45	5.08	4.72
Part 2 POST	11.00	7.348	14.69	4.97	7.08	6.85	3.67	6.31
Overall POST	42.83	18.46	51.38	12.48	35.75	22.21	29.17	17.69
Warm up DIF	3.33	4.29	2.00	4.637	1.08	1.83	−2.83	5.11
Part 1 DIF	6.58	8.27	6.46	6.61	4.42	6.47	−1.00	8.71
TC DIF	0.75	4.01	3.31	3.49	1.75	3.38	−1.67	5.58
Part 2 DIF	6.58	8.18	5.77	5.41	1.17	5.31	−1.42	4.62
Overall DIF	17.67	11.91	18.77	11.88	8.58	9.25	−6.83	14.71
Part 1 CCE	4.08	3.81	4.08	3.52	2.42	3.63	0.00	4.26
TC CCE	0.58	2.11	1.31	1.88	1.00	2.00	−0.75	2.83
Part 2 CCE	3.00	3.21	3.31	1.93	0.00	1.91	−0.67	2.23
Overall CCE	7.67	4.49	8.69	5.25	3.42	4.88	−1.42	5.36

Note: SD: Standard deviation; GDC: General dynamic coordination; TC: task change; CCE: Cognitive control estimation; DIF: differential score obtained by post score minus pre score.
